# Selecting Tolerant Maize Hybrids Using Factor Analytic Models and Environmental Covariates as Drought Stress Indicators

**DOI:** 10.3390/genes16070754

**Published:** 2025-06-27

**Authors:** Domagoj Stepinac, Ivan Pejić, Krešo Pandžić, Tanja Likso, Hrvoje Šarčević, Domagoj Šimić, Miroslav Bukan, Ivica Buhiniček, Antun Jambrović, Bojan Marković, Mirko Jukić, Jerko Gunjača

**Affiliations:** 1 Bc Institute for Breeding and Production of Field Crops, Dugoselska 7, 10370 Dugo Selo, Croatia; domagoj.stepinac@bc-institut.hr (D.S.); ibuhinicek@bc-institut.hr (I.B.); mjukic@bc-institut.hr (M.J.); 2 University of Zagreb, Faculty of Agriculture, Svetošimunska cesta 25, 10000 Zagreb, Croatia; ipejic@agr.hr (I.P.); hsarcevic@agr.hr (H.Š.); mbukan@agr.hr (M.B.); 3 Croatian Meteorological and Hydrological Service, Ravnice 48, 10000 Zagreb, Croatia; kresho.pandzic@gmail.com (K.P.); likso@cirus.dhz.hr (T.L.); 4 Agricultural Institute Osijek, Južno Predgrađe 17, 31000 Osijek, Croatia; domagoj.simic@poljinos.hr (D.Š.); antun.jambrovic@poljinos.hr (A.J.); 5 Croatian Agency for Agriculture and Food, Centre for Seed and Seedlings, Usorska 19, 31000 Osijek, Croatia; boker031@gmail.com

**Keywords:** maize, drought tolerance, scPDSI (Self-calibrating Palmer Drought Severity Index), VPD (Vapor Pressure Deficit), abiotic stress, grain yield stability

## Abstract

**Background/Objectives**: A critical part of the maize life cycle takes place during the summer, and due to climate change, its growth and development are increasingly exposed to the irregular and unpredictable effects of drought stress. Developing and using new cultivars with increased drought tolerance for farmers is the easiest and cheapest solution. One of the concepts to screen for drought tolerance is to expose germplasm to various growth scenarios (environments), expecting that random drought will occur in some of them. **Methods**: In the present study, thirty-two maize hybrids belonging to four FAO maturity groups were tested for grain yield at six locations over two consecutive years. In parallel, data of the basic meteorological elements such as air temperature, relative humidity and precipitation were collected and used to compute two indices, scPDSI (Self-calibrating Palmer Drought Severity Index) and VPD (Vapor Pressure Deficit), that were assessed as indicators of drought (water deficit) severity during the vegetation period. Practical implementation of these indices was carried out indirectly by first analyzing yield data using a factor analytic model to detect latent environmental variables affecting yield and then correlating those latent variables with drought indices. **Results**: The first latent variable, which explained 47.97% of the total variability, was correlated with VPD (r = −0.58); the second latent variable explained 9.57% of the total variability and was correlated with scPDSI (r = −0.74). Furthermore, latent regression coefficients (i.e., genotypic sensitivities to latent environmental variables) were correlated with genotypic drought tolerance. **Conclusions**: This could be considered an indication that there were two different acting mechanisms in which drought affected yield.

## 1. Introduction

Maize is an annual spring crop produced worldwide. As its biological cycles occur during the warmest part of the year, maize crops are increasingly exposed to drought episodes. Global annual losses in maize yields due to drought have been estimated at around 15%, equivalent to damages of around USD 36 billion [1]. The IPCC [2] projects even worse future impacts of climate change by 2100.

For the development of promising breeding populations, leading cultivars (their inbred lines) are usually used. Phenotyping efforts are primarily focused on grain yield as a principal trait. Development of drought-tolerant maize hybrids by classical breeding methods relies on the existence of sufficiently large genetic variation in germplasm, performing well both under stressful and normal conditions. More precisely, the success of breeding for drought tolerance, besides the adequate germplasm, also depends on the availability of a series of environments (test conditions) in which the intensity of stress and its timing can be controlled or monitored and their influence on trait expression during breeding cycles [3]. Therefore, Xu [4] and Yue et al. [5] propose the envirotyping (environmental typification) concept to select specific environmental sites that contribute through their functional components to understanding genotype-by-environment interaction (GEI) and genes’ response to environmental signals regarding biotic and abiotic stresses such as drought. Along with precise envirotypic, genotypic and phenotypic information, it is possible to establish a highly efficient precision breeding and sustainable crop production system [4]. On the other hand, due to the limited availability of such systems and environments, most breeding programs focus only on increasing productivity under favorable conditions where the success of selection for yield is highest. The inclusion of random drought environments in multi-environmental experiments is often the only way to increase the efficiency of breeding for drought tolerance [6].

Environment characterization can be performed by using the Self-calibrating Palmer Drought Severity Index (scPDSI) [7,8,9,10,11,12] and Vapor Pressure Deficit (VPD) [13,14,15,16].

The scPDSI is an index whose value reflects the deviation of the current weather status from the general climatic characteristics of the test site. The scPDSI values directly depend on seasonal air temperatures (month or decade averages) and corresponding precipitation, as well as their historical values for the test location [12]. Even though it was originally developed for other purposes (meteorology, hydrology), this index has proven useful in detecting and assessing agricultural drought [10,11,12]. Its values are probably a good indicator of the status of soil water content and water availability to the root system.

The VPD index is calculated from air temperature and relative humidity. VPD is a critical measure of the atmospheric demand for water and can be used to assess short-term and seasonal drought [17]. Its value has been shown to be related to the efficiency of photosynthesis and respiration processes [18]. High VPD values caused by high air temperatures over a longer period are associated with slowed daily growth, but the rate is genotype specific [14,19].

On the other hand, genotype reaction to drought is often measured and estimated by the level of trait value reduction (difference between stress and non-stress environments). For simple genotype selection and ranking, plant breeders of various species use various drought-tolerance indices. Usually, these indices are calculated from data collected in contrasting treatments, most often intentionally induced at one location. Depending on the formula, they express the average productivity or the difference in the value of the same genotype between stressful and favorable conditions [20,21,22,23,24,25,26]. However, the stage of plant development at which stress occurs can greatly influence the value of the index of the same genotype.

Factor-analytic models (FAs) are mixed models analogous to AMMI [27], popular and widely used models for the analysis of GEI. The main advantage of FAs is the provision of the set of tools for the interpretation of GEI, coined “Factor analytic selection tools” (FAST) by Smith and Cullis [27]. They were applied in the analysis of maize METs for the genomic prediction for drought tolerance [28], pro-vitamin A content and yield in maize synthetics [29], genomic prediction for yield [30] and selection of high-performance and stable tropical hybrids [31]. If the FAs’ latent factors could be related to environmental covariates, it would provide the possible biological interpretation of the GEI [32].

The present research was based on field trials involving 32 commercial maize hybrids sown at six locations in the continental part of Croatia over two consecutive seasons. Collected data includes yield and other agronomical traits as well as meteorological data for the trial locations. Assuming that it would be possible to relate latent variables with environmental covariates, the objectives of the present research were to (i) find the optimal FA for the analysis of maize yield data, (ii) correlate latent factors with environmental covariates, and (iii) identify stable and stress-tolerant hybrids.

## 2. Materials and Methods

Field trials with maize hybrids were set at 6 rainfed locations in continental Croatia (Beli Manastir, Kutjevo, Osijek, Rugvica, Šašinovec and Tovarnik, during two consecutive years (2017–2018) (Table S1). Hybrids were selected from 4 different maturity groups (FAO300, FAO400, FAO500 and FAO600), and each group was represented with 8 hybrids, giving a total of 32 hybrids (Table S2). The trial design was a row-column design generated using CycDesigN 8 software [33]. Layouts were randomized for each group separately, giving a total of 48 trials (6 locations × 2 years × 4 maturity groups). For each experimental plot, the grain yield at 13% moisture (GY) has been recorded.

Based on the meteorological data, such as air temperature, precipitation and air humidity, measured in an hourly regime for the vegetation period (May to September) by the automated weather station placed within the field experiments, scPDSI and VPD 10 days/monthly drought stress indices were computed [12,13]. The status of drought stress of each environment (location × year) as measured by scPDSI is expressed by a numerical value that can be interpreted by an existing scale [7]. The values of VPD are expressed in kPa. According to Gholipoor et al. [34], values between 1.7 and 2.5 kPa affected the transpiration rate of many maize hybrids, and Choudhary et al. [15] found the same for values above 3 kPa. Higher values of air VPD correlate well with increases in drought stress.

The average location ScPDSI and VPD values were used to select 3 normal and 3 most severe drought environments. Based on the average yields of individual hybrids in two contrasting environments, Geometric Mean Productivity (GMP) [21] and Relative Decrease in Yield (RDY) [24] drought-tolerance indices were computed for each hybrid.

Analysis of the yield data was carried out in two steps. The first step consisted of the analyses of individual trials, starting with searching for the optimal model within the collection of 640 predefined models in the R package ‘ASRtriala’ (Version 1.0.0) [35], an add-on package for the ‘ASReml-R 4.2’ [36]. The effect of hybrids was treated as a random effect, so the search was carried out using the generalized heritability as goodness-of-fit criteria [37]. After finding the optimal model, the data were refitted to the model with the fixed effect of hybrids to obtain un-shrunken predictions of genotypic yield [38,39]. Obtained predictions and their standard errors were then extracted to be used as the input for the second step, the MET (Multi-Environment Trial) analysis. The second step started with another search for the optimal model. This time it is the selection of the optimal MET model from the five GEI nested models (factor analytic models fa1 to fa5), using the AIC as the selection criteria. Selected model predictions and parameters were then used in the interpretation of the GEI patterns, assisted by various plots created as the standard output of the R package ‘ASRtriala’. Finally, seeking the biological interpretation of the hypothetic environmental and genotypic covariates (fitted model outputs), they are correlated with stress and tolerance indices [32].

## 3. Results

### 3.1. Environmental Characterization

**Figure 1:**
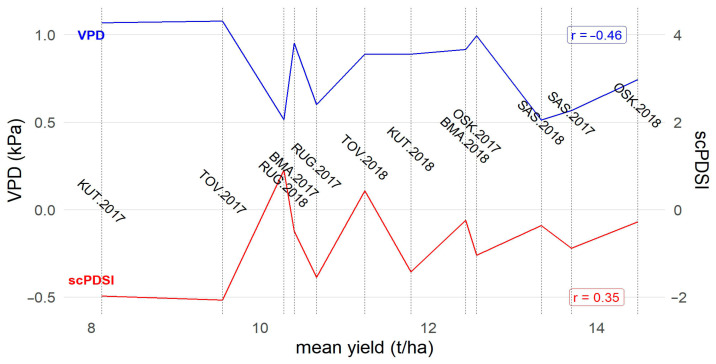
The relationship between average yields and water deficit indexes VPD (Vapor Pressure Deficit) and scPDSI (Self-calibrating Palmer Drought Severity Index).

After fitting the optimal models for single trials, yield predictions and their standard errors for hybrids from all four maturity groups were all pooled together. Summary statistics for all the environments (location × year combinations) are presented in Table S1. Average yields over all four maturity groups ranged from 8.12 t/ha (KUT.2017) to 14.49 t/ha (OSK.2018). Yields were generally higher in 2018, except for Rugvica and Šašinovec, where there was only a small difference between years (with slightly higher yields in 2017). For all locations except Šašinovec, the relative variation in the genotypic estimates was either slightly higher in 2017 or similar in both years. At Šašinovec, the difference between mean yields was small, but relative variation in genotypic estimates is almost twofold higher in 2018. Based on measured meteorological data and derived index (scPDSI and VPD) values, as well as their comparisons with multiyear data, 2017 showed to be a moderately dry year and 2018 a nearly normal year. The occurrence of water deficit in 2017 and its impact on yield can be further elucidated by comparing drought stress indices VPD and scPDSI with the average yields (Figure 1).

According to the scPDSI, the presence of water deficit (scPDSI < −0.49) was detected at all locations in 2017, as well as in Kutjevo in 2018. All other locations in 2018 had normal conditions, with even a slight water surplus at Rugvica. The interpretation of VPD values is less straightforward, but in this case high values indicate the presence of water deficit. The most obvious disagreement between indices in 2017 is observed at the western locations Rugvica and Šašinovec; VPD identifies them as normal environments, but scPDSI indicates the occurrence of water deficit. Correlations between average yields and both indexes were weak to average, with a more pronounced trend for VPD. However, the two lowest yielding environments (Kutjevo and Tovarnik in 2017) were also identified as the most stressful ones according to both indexes. If there was a water deficit at western locations in 2017 (as scPDSI suggests), it did not affect yields, because the difference between years at these two locations is quite small (and yields were even higher in 2017).

### 3.2. MET Analysis

Goodness-of-fit statistics for the five fitted MET models are shown in Table 1. According to AIC, the best MET model is fa2. The more complex factor-analytic models (fa3 and fa4) were more efficient, but they were penalized for the expense of additional model parameters, thus resulting in a higher AIC value. Therefore, fa2 model was selected as a basis for the interpretation of GEI.

**Figure 2:**
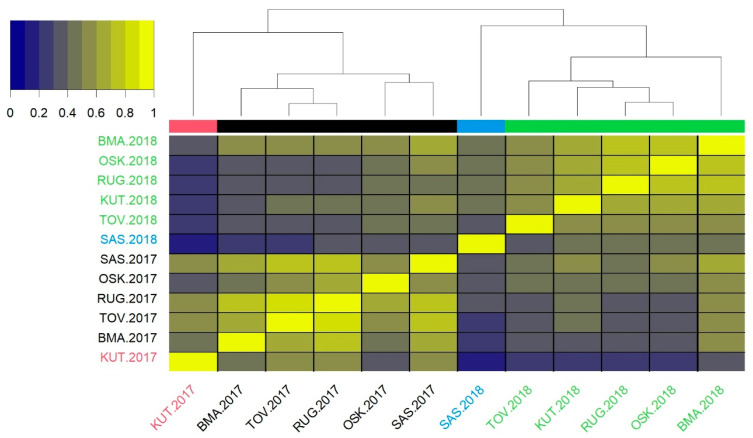
Heatmap of pairwise genetic correlations between environments. Clusters at the four-cluster solution are designated by colors (red, black, blue and green).

All genetic correlations between environments have positive values (Figure 2). There is a simple general pattern, as all environments are grouped by years. Kutjevo in 2017 and Šašinovec in 2018 stand out from all other locations in the respective year; thus, in a four-cluster solution, they form the clusters on their own. The correlation between Kutjevo 2017 and Šašinovec 2018 is the weakest one (0.20). Their correlations with the rest of the environments are stronger but do not exceed 0.60. Correlations between environments in 2017 (excluding Kutjevo) range from 0.46 to 0.81, while correlations between environments in 2018 (excluding Šašinovec) range from 0.51 to 0.78. This indicates the general pattern of occurrence of low GEI within years followed by stronger GEI between years. The exceptions are Kutjevo 2017 and Šašinovec 2018, with relatively strong GEI towards all other environments and the strongest GEI between them.

Two factors of the selected best model (fa2) cumulatively explained 57.54% of the total variability (47.97% and 9.57%, respectively). For all locations, loadings for the first factor are higher in 2018, except for Osijek and Tovarnik (Table 2). Loadings of the second factor have positive signs in 2017 and negative signs in 2018. The largest portion of the genetic variance is explained by the fa2 model at Rugvica (in both years), while Kutjevo 2017 and Šašinovec 2018 are environments in which most of the genetic variation is left unexplained.

### 3.3. Yield Stability

**Figure 3:**
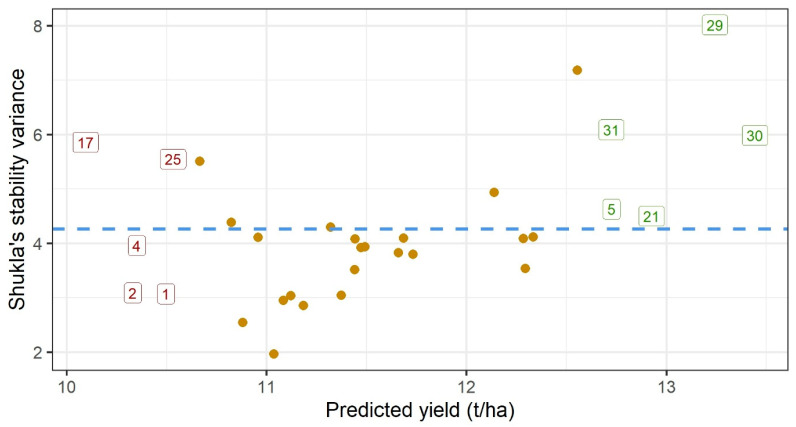
Predicted yields vs. yield stability (Shukla’s variance) of the 32 maize hybrids. Five top-yielding hybrids are labeled green; five bottom-yielding hybrids are labeled red. All the remaining hybrids are designated by yellow dots. The blue dashed line designates average stability.

A quick way to identify favorable genotypes is to plot their predicted yields vs. yield stability (Figure 3). High-yielding genotypes are positioned on the right-hand side of the plot (labeled green). They predominantly belong to the later maturity groups, except for hybrid no. 5 (from FAO 300). Three of the five top yielding hybrids are quite unstable, thus leaving nos. 5 and 21 to be selected as the high-yielding and relatively stable hybrids.

**Figure 4:**
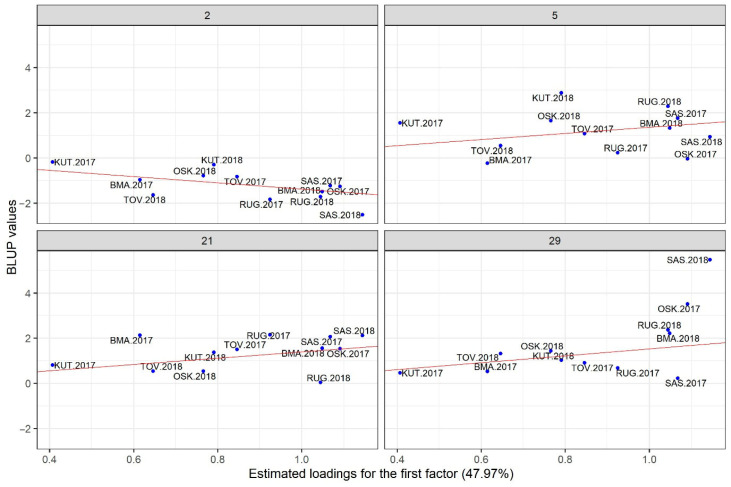
Latent regression plots for the hybrids 2, 5, 21 and 29 using the first factor (the solid red line is the latent regression line).

**Figure 5:**
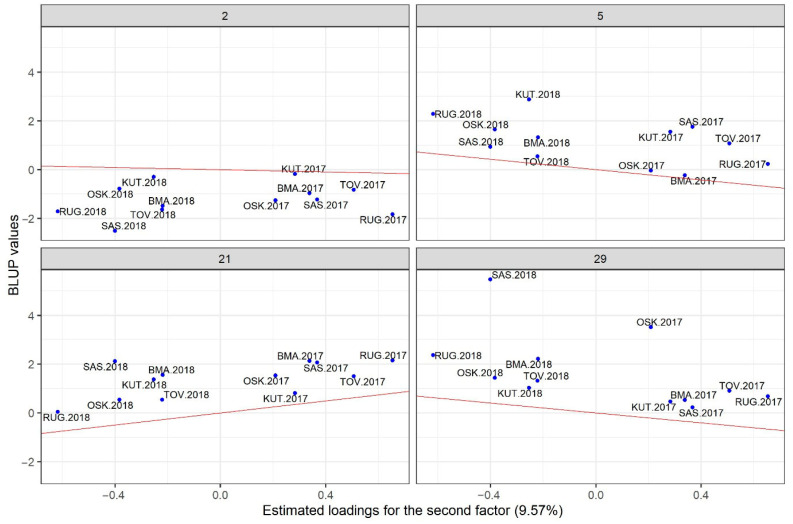
Latent regression plots for the hybrids 2, 5, 21 and 29 using the second factor (the solid red line is the latent regression line).

Closer insight into the performance of each of the most promising genotypes can be obtained by plotting their BLUPs against the factor loadings. In Figure 4 and Figure 5, the two most interesting hybrids (5 and 21) are compared with one high-yielding but most unstable hybrid (29) and one stable but low-yielding hybrid (2). Five top-yielding hybrids (labeled green on Figure 3) also have the highest positive slopes of regression on the first factor. They are highly sensitive to environmental improvements; therefore, they are adapted to the environments with the high loadings for the first factor. Figure 4 reveals that hybrids 5, 21 and 29 are adapted to environments on the right-hand side of the plot (high yielding), while hybrid 2 is adapted to environments on the left-hand side of the plot (low yielding). Points for three stable hybrids are clustered relatively tightly around the regression line, but there is a large spread of points on the right-hand side of the plot for the unstable hybrid 29. Consequently, the selected model will be less accurate in predicting the performance of hybrid 29 compared to hybrids 2, 5 and 21. Latent regression plots for the second factor (Figure 5) provide insight into the sensitivities of hybrids to this latent factor. Observed patterns differ from those described for the first factor: hybrid 2 is quite insensitive to the second factor (slope close to zero), hybrid 21 again has a positive slope, while hybrids 5 and 29 have negative slopes.

### 3.4. Correlations with Environmental and Genotypic Covariates

Pearson’s correlation coefficients between environmental factor loadings (for the factors FA1 and FA2) and environmental covariates are given in Table 3. The strongest correlations with the first factor were observed for the relative humidity and VPD. Therefore, these two covariates were most likely candidates for explaining GEI for maize yield: hybrids more sensitive to the first latent factor are at the same time more sensitive to relative humidity and VPD. scPDSI is the covariate with the strongest correlation to the second latent factor; thus, the hybrids more sensitive to the second factor are also more sensitive to scPDSI.

For each hybrid, two drought-tolerance indices (GMP and RDY) have been calculated (Table S3). A lower value of the GMP index indicates greater tolerance to drought, while the reverse is true in the case of the RDY index. However, each of the indices singles out different hybrids as tolerant, as their mutual correlation is very weak (r = 0.06); therefore, they indicate different elements of tolerance.

Similarly to correlating factors with environmental covariates, hybrid latent regression slopes can be correlated with drought tolerance indices (Table 4). Regression slopes are hybrid sensitivities to latent factors; therefore, sensitivity to the first factor can be associated with GMP, while sensitivity to the second factor can be associated with RDY.

## 4. Discussion

The reliability of the two-stage approach in the analysis of METs depends upon the fulfillment of the prerequisites described by Smith et al. [40]. As they stress out, that is “particularly important when there is substantial heterogeneity of within-trial error variances”, like greater than five ratios of max to min variance in the present study (as reported by ‘ASRtriala’). The use of optimal models for the analysis of individual trials can improve the accuracy of the hybrid’s predictions and their weights (to be carried through to the second stage). Unless some method of automation is used, a search for the optimal model can be a laborious task. A run through the collection of 640 predefined models in ‘ASRtriala’ took less than a minute (bearing in mind that the size of each individual trial was relatively small—32 plots).

Considering the set of locations used in the present study, more or less pronounced water deficit was detected at all of them in 2017, while 2018 was a relatively normal year. The two most severely affected environments, Kutjevo 2017 and Tovarnik 2017, can be classified as ‘slightly dry’ and ‘moderately dry’, respectively [7]. Western locations Rugvica and Šašinovec represent the exception from the general pattern: a small difference between years with slightly higher yields in 2017 and normal environments according to VDP vs. indicated water deficit by scPDSI.

The selected MET model (fa2) explained 57.54% of the total variability, which means that there is a considerably large part of the variability left unexplained by the model. Factor analytic models could be more efficient, as reported by Chaves et al. [31]. They have achieved 70–79% variability by the models fa3 and fa4, using a larger dataset of 60 genotypes grown in two years at 27 locations. However, looking at the individual environments, it can be concluded that a relatively modest amount of explained variance in the present study is mostly due to extremely low values in two misfitted environments (Kutjevo 2017 and Šašinovec 2018), characterized by strong crossover GEI with the rest of the environments. Outgrouping of these two environments suggested clustering environments into four groups, in which they form separate groups of their own, while all other environments are grouped by the years. Therefore, the separation of stress and non-stress environments was not clear, as reported in some other GEI studies [41].

One of the positive aspects of the fitted model in the present study is that all factor loadings for the first factor have positive signs, which means that fitted values related to the first factor represent non-crossover interaction. This is an important prerequisite for the use of predicted value vs. stability plots for the selection of promising genotypes [27]. Selection of the hybrids based on Figure 3 represents general recommendations across all environments. In addition to that, Chaves et al. [40] also provide specific recommendations for each of the seasons in their study. The same approach could not be followed in the present study, mainly due to the presence of two outgrouped environments. However, selected hybrids could be generally recommended, as they performed well even in unfavorable environments. Recommendations based on model predictions should be taken with caution only when they refer to unstable hybrids, especially in outgrouped environments. Three of the four highlighted hybrids (Figure 4 and Figure 5) correspond well with the general observation that early maturity hybrids are better adapted to dry conditions [42,43].

Correlations of factor loadings with environmental covariates provide a means for their biological interpretation. Both selected model factors could be associated with water deficit, as they were correlated with two drought indexes, VPD and scPDSI (r = −0.58 and r = −0.74, respectively). Hybrids latent regression coefficients can be associated with their drought tolerance indexes; thus, their response to changes in environmental conditions reflects their drought tolerance. It could then be further speculated that sensitivity to VPD is somehow associated with GMP, and sensitivity to scPDSI is somehow associated with RDY. VPD was detected as one of the two most important environmental covariates contributing to GEI observed on maize yield under heat stress by Madhumal Thayil et al. [44], who fitted the environmental covariates directly using the factorial regression models. Their second most important covariate was relative humidity; in our study, the correlation between the first factor and relative humidity was equally as strong as the correlation with VPD. It could be noted that they were using VPD and relative humidity interchangeably through the different reproductive stages, while we used them summarily over the whole season.

When ordered by their average yields, environments from 2017 are intertwined with environments from 2018 even though water deficit was more pronounced in 2017. This creates the specific sawtooth wave form of lines on Figure 1, pointing out that there is still 42.46% of variability that should be explained by other environmental factors. Recently, several studies by other authors applied FAs in the analysis of METs and reported weak to strong correlations between factor loadings and environmental covariates. Oliveira et al. [32] analyzed sorghum fresh biomass yield; the strongest correlation that they found was between second factor loadings and altitude (r = 0.45). Coulibaly et al. [45] analyzed Kersting’s groundnut yield and two other traits; they found very strong correlations with precipitation, temperature and humidity (|r| = 0.80 to 0.97). Filho et al. [46] analyzed cassava’s yield traits and found the strongest correlation with rainfall (r = 0.58) and altitude (r = 0.55). Better correspondence with the FA could be achieved by direct inclusion of environmental covariates through the integrated factor analytic linear mixed model (IFA-LMM) developed by Tolhurst et al. [47]. However, that would require the availability of a more comprehensive set of environmental covariates.

## 5. Conclusions

Factor analytic models could provide a convenient tool for selecting stress-tolerant genotypes, especially when detected latent variables can be correlated to environmental covariates. However, recommendations should be made with some caution for the individual environments that express strong crossover GEI with the rest of the environments that were misfitted by the selected optimal model. Those environments would require specific recommendations that would not follow the general rule.

## Figures and Tables

**Table 1:** Goodness-of-fit statistics for fitted MET models.

Model	No. of Var. Comp.	logLik	AIC
fa1	24	−214.93	477.86
fa2	35	−194.68	459.35
fa3	43	−187.81	461.62
fa4	51	−180.11	462.22
fa5	59	−174.09	466.18

**Table 2:** Rotated environmental factor loadings for fa2 model and cumulative % of the genetic variance explained for each environment.

Location	Year	FA1	FA2	Cum. % Gen. Var.
Beli Manastir	2017	0.61	0.34	56.01
	2018	1.05	−0.22	78.87
Kutjevo	2017	0.41	0.28	38.15
	2018	0.79	−0.25	57.45
Osijek	2017	1.09	0.21	49.10
	2018	0.77	−0.38	75.76
Rugvica	2017	0.92	0.65	90.35
	2018	1.04	−0.62	80.88
Šašinovec	2017	1.07	0.37	72.08
	2018	1.14	−0.40	29.92
Tovarnik	2017	0.85	0.51	72.77
	2018	0.65	−0.22	45.05

**Table 3:** Correlations between factor loadings and environmental covariates.

Covariate	FA1	FA2
T_min_ (°C)	−0.44	−0.11
T_max_ (°C)	−0.32	0.14
T_avg_ (°C)	−0.44	−0.10
Rainfall (mm)	0.41	−0.22
Relative humidity (%)	0.59	−0.33
VPD	−0.58	0.34
scPDSI	0.27	−0.74

**Table 4:** Correlations between hybrid tolerance indexes and slopes.

Index	β_1_ (FA1)	β_2_ (FA2)
GMP	0.95	0.03
RDY	0.05	−0.73

## Data Availability

Dataset available on request from the authors.

## Supplementary Materials

The following supporting information can be downloaded at https://www.mdpi.com/article/10.3390/genes16070754/s1, Table S1: Information on field experiment locations and their summary statistics; Table S2: List of maize genotypes (single-cross hybrids) used for field trials; Table S3: Values of the Geometric Mean Productivity (GMP) and Relative Decrease in Yield (RDY) drought-tolerance indices for 32 tested maize hybrids.
