# Supplementary material for: Selecting Tolerant Maize Hybrids Using Factor Analytic Models and Environmental Covariates as Drought Stress Indicators
Source: Genes (Basel). 2025 Jun 27;16(7):754. doi: 10.3390/genes16070754 (PMC12294160; doi:10.3390/genes16070754)
Supplement: Supplementary file 1 [file genes-16-00754-s001.zip › genes-3695583-supplementary.pdf]

Table S1. Information on field experiment locations and their summary statistics

Location	GPS	Year	Environment code	Mean yield (t/ha)	CV (%)
Šašinovec	45°51'09.7"N	2017	SAS.2017	13.7	10.25
	16°10'49.4"E	2018	SAS.2018	13.34	17.16
Rugvica	45°45'13.4"N	2017	RUG.2017	10.67	12.42
	16°14'31.8"E	2018	RUG.2018	10.28	13.79
Kutjevo	45°24'28.0"N	2017	KUT.2017	8.12	10.00
	17°53'18.1"E	2018	KUT.2018	11.79	9.85
Beli Manastir	45°48'09.4"N	2017	BMA.2017	10.41	9.66
	18°39'08.9"E	2018	BMA.2018	12.44	9.96
Osijek	45°32'11.7"N	2017	OSK.2017	12.57	12.85
	18°44'28.1"E	2018	OSK.2018	14.49	7.84
Tovarnik	45°10'17.0"N	2017	TOV.2017	9.56	12.21
	19°08'25.6"E	2018	TOV.2018	11.24	9.22

Table S2. List of maize genotypes (single-cross hybrids) used for field trials

Code	Name of genotype	FAO maturity group	Breeding company
1	BC344	300	Bc Institut, d.d., Croatia
2	BC306	300	Bc Institut, d.d., Croatia
3	BC323	300	Bc Institut, d.d., Croatia
4	TRIANGLE	300	Bc Institut, d.d., Croatia
5	P9903	300	Pioneer Hi-Bred Services GmbH Industriegelande; Corteva Agriscience Croatia d.o.o.
6	Os378	300	Agricultural Institute Osijek, Croatia
7	Os398	300	Agricultural Institute Osijek, Croatia
8	Os3617	300	Agricultural Institute Osijek, Croatia
9	OS444	400	Agricultural Institute Osijek, Croatia
10	BC406	400	Bc Institut, d.d., Croatia
11	BC424	400	Bc Institut, d.d., Croatia
12	BC482	400	Bc Institut, d.d., Croatia
13	DKC4608	400	Bayer Seeds SAS; Bayer d.o.o.
14	Kulak	400	Agricultural Institute Osijek, Croatia
15	Tomasov	400	Agricultural Institute Osijek, Croatia
16	Drava404	400	Agricultural Institute Osijek, Croatia
17	OS552	500	Agricultural Institute Osijek, Croatia
18	BC525	500	Bc Institut, d.d., Croatia
19	BC575	500	Bc Institut, d.d., Croatia
20	KLIPAN	500	Bc Institut, d.d., Croatia
21	DKC5830	500	Bayer Seeds SAS; Bayer d.o.o.
22	Velimir	500	Agricultural Institute Osijek, Croatia
23	Os5922	500	Agricultural Institute Osijek, Croatia
24	OS515	500	Agricultural Institute Osijek, Croatia
25	BC682	600	Bc Institut, d.d., Croatia
26	BC616	600	Bc Institut, d.d., Croatia
27	BC626	600	Bc Institut, d.d., Croatia
28	RIĐAN	600	Bc Institut, d.d., Croatia
29	P1535	600	Pioneer Hi-Bred Services GmbH Industriegelande; Corteva Agriscience Croatia d.o.o.
30	Rudolf	600	Agricultural Institute Osijek, Croatia
31	Os6217	600	Agricultural Institute Osijek, Croatia
32	Os635	600	Agricultural Institute Osijek, Croatia

Table S3. Values of the Geometric Mean Productivity (GMP) and Relative Decrease in Yield (RDY) drought-tolerance indices for 32 tested maize hybrids

Hybrid (Genotype) Code	GMP	RDY
1	10.43	13.80
2	10.03	16.40
3	10.68	4.60
4	9.88	12.90
5	12.42	25.20
6	10.71	9.10
7	10.91	15.40
8	10.89	20.10
9	10.05	39.70
10	11.2	15.70
11	10.55	7.60
12	11.91	17.60
13	11.36	14.10
14	12.08	19.20
15	11.8	15.10
16	10.79	17.70
17	10.12	33.50
18	11.21	17.40
19	11.26	21.90
20	10.54	21.40
21	12.48	19.00
22	11.33	22.80
23	11.88	36.10
24	10.46	29.20
25	10.17	38.20
26	11.08	28.30
27	11.4	21.50
28	11.24	27.00
29	13.54	27.60
30	13.28	19.40
31	12.69	25.50
32	11.74	25.10
